# Guideline adherence in depression outcome measurement: a retrospective cohort study from Swedish psychiatric outpatient care

**DOI:** 10.3389/fpsyt.2025.1667897

**Published:** 2025-11-03

**Authors:** Samir El Alaoui, Nitya Jayaram-Lindström, Benjamin Bohman

**Affiliations:** Centre for Psychiatry Research, Department of Clinical Neuroscience, Karolinska Institutet, & Stockholm Health Care Services, Region Stockholm, Stockholm, Sweden

**Keywords:** measurement-based care, routine outcome monitoring, depression, digital mental health, internet-based cognitive behavioral therapy, implementation science, electronic health records

## Abstract

**Background:**

Routine outcome measurement is a core element of measurement-based care (MBC), yet its use in everyday psychiatric practice remains limited. Even minimal follow-up assessment is often missing despite explicit clinical guideline recommendations.

**Methods:**

We conducted a retrospective cohort study of 2431 adult outpatient episodes of depression treatment within Stockholm’s public mental health system (2020–2023). Guideline-concordant outcome measurement was defined as documentation of at least one Patient Health Questionnaire-9 (PHQ-9) or clinician-rated Montgomery–Åsberg Depression Rating Scale (MADRS) within 60 days of treatment initiation for all modalities, or within ten sessions for psychotherapy. Adherence rates were compared across antidepressant pharmacotherapy, face-to-face psychotherapy, and internet-based cognitive behavioral therapy (iCBT).

**Results:**

Among treatment episodes lasting ≥ 30 days (n = 2242), 28.2% included a PHQ-9 or MADRS within 60 days. Adherence was higher in psychological treatments (71.6%) than in pharmacotherapy (10.2%), χ²(1, N = 2242) = 865.14, *p* <.001, Cramer’s *V* = .62. Within psychotherapy, iCBT showed markedly greater adherence (80.1%) than traditional psychotherapy (18.0%), χ²(1, N = 656) = 146.00, *p* <.001, *V* = .47. When iCBT was excluded, adherence fell to 10.6% overall. Among psychotherapy episodes with ≥ 10 sessions (n = 482), 73.4% met the ten-session guideline, with 79.5% adherence in iCBT and 23.1% in traditional psychotherapy, χ²(1, N = 482) = 75.82, *p* <.001, *V* = .40. Younger clinicians (M = 39.8 vs. 46.8 years, *p* <.001) and psychologists (73%) showed higher adherence than physicians (10%) or other professions.

**Conclusion:**

Even under conservative criteria, minimal outcome measurement was documented in fewer than one-third of depression treatment episodes. Adherence was particularly low in pharmacotherapy and traditional psychotherapy but substantially higher in iCBT, reflecting the advantages of automated, integrated digital workflows. These findings underscore that policy guidelines alone are insufficient; scalable implementation of MBC requires integrated digital systems, structured workflows, and targeted clinician support.

## 1 Introduction

Measurement-based care (MBC)—the systematic use of validated outcome measures to inform clinical decision-making—has consistently been associated with improved outcomes across psychiatric and behavioral health settings. Randomized trials such as STAR*D (1) and subsequent meta-analyses have shown that MBC reduces symptom severity, increases remission rates, improves adherence, and lowers the risk of deterioration, particularly in patients not progressing as expected (2–5). Recent reviews emphasize that the systematic use of patient progress feedback is central to achieving these benefits and represents the benchmark for high-quality MBC (6). Despite this evidence, routine uptake of MBC remains limited internationally: surveys indicate that only a minority of clinicians regularly use standardized outcome measures in everyday care (7, 8). Common barriers include time burden, insufficient training, and skepticism regarding clinical utility (5, 9).

Sweden provides a distinctive case for examining MBC implementation. As part of a regional reform program, Stockholm Health Care Services (SHCS) introduced standardized care process charts to promote evidence-based and equitable psychiatric care (10). These charts specify the use of structured outcome measures such as the Montgomery–Åsberg Depression Rating Scale (MADRS) (11) and the Patient Health Questionnaire-9 (PHQ-9) (12) at intake and during treatment. They require at least one documented assessment within 60 days of treatment initiation and, for psychotherapy, within the first ten sessions (13). These represent formally endorsed policy expectations, supported through EHR templates and digital web forms. However, integration into clinical workflows varies across clinics, and adherence is monitored mainly for quality improvement rather than enforced through audits or sanctions.

A parallel development in Sweden has been the nationwide implementation of digital mental health services (DMHS) through the Support and Treatment platform on 1177.se. This system enables patients to log in securely and access structured digital programs for psychological support, education, and treatment. Among these, internet-based cognitive behavioral therapy (iCBT) for depression and anxiety is the most widely used and represents Sweden’s primary DMHS model for structured, therapist-guided psychological care (14, 15). Programs are delivered by licensed healthcare providers and typically include weekly therapist contact via secure messaging or brief video sessions. This model, integrated into specialized psychiatry, combines clinical quality with scalability and accessibility, offering evidence-based treatment with minimal disruption to patients’ daily routines (15, 16). The Swedish implementation thus mirrors international DMHS frameworks (e.g., Australia, the UK), where digital platforms provide standardized, evidence-based treatment modules supported by automated symptom monitoring and clinician feedback systems. Within this infrastructure, iCBT represents both the most mature and most widely adopted example of DMHS in specialized psychiatric care.

The rapid expansion of iCBT reflects both clinical demand and explicit policy initiatives. In 2016, the Swedish Government and the Swedish Association of Local Authorities and Regions launched *Vision for eHealth 2025*, aiming to make Sweden a world leader in eHealth (17). The strategy for implementing this vision emphasized positioning the individual as a co-creator of care, ensuring access to reliable knowledge, guaranteeing safe information management, and aligning digital transformation with organizational development (18). Progress reports note substantial advances—such as nationwide deployment of the 1177 portal and expanded access to digital treatment programs—alongside persistent challenges, including regional disparities, interoperability gaps, and information security demands (19). Within this framework, digital mental health interventions such as iCBT have been prioritized to expand access, standardize care workflows, and enhance patient participation. Region Stockholm also operates the Internet Psychiatry Clinic, which accepts nationwide referrals, while 1177 serves as the national entry point for digital health services. Within this system, iCBT is delivered by licensed psychologists and psychotherapists working in specialized psychiatric services, ensuring that digital treatment meets the same clinical and regulatory standards as face-to-face care. Controlled studies have shown that iCBT achieves outcomes equivalent to traditional psychotherapy while offering lower societal costs through reduced travel time, minimal productivity loss, and high accessibility (14, 15, 20). These initiatives—grounded in equity and efficiency goals—have positioned Sweden as a frontrunner in the integration of iCBT into routine psychiatric care. However, there are currently no formal financial or professional incentives for clinicians to engage in iCBT delivery, which may limit broader uptake despite demonstrated clinical effectiveness.

Experiences from large-scale digital mental health services in Australia similarly indicate that therapist-supported iCBT can be delivered safely and effectively at population scale, with routine outcome measurement embedded in care and outcomes comparable to face-to-face CBT (16). Over a decade of service delivery, national clinics reported high volumes, consistent symptom improvement, and robust governance processes, underscoring how digital platforms can operationalize routine measurement in everyday care. These international experiences provide a useful counterpoint and motivation for examining guideline-concordant documentation within Swedish services.

Digital infrastructure also underpins outcome measurement in traditional psychiatric care. In Region Stockholm and several other regions, outcome assessments in pharmacotherapy and face-to-face psychotherapy are typically administered through the *Web forms* system, which is integrated with the EHR. When patients complete questionnaires via this system, results can be reviewed in advance and, once saved by the clinician, automatically stored as structured data in the EHR. In contrast, iCBT delivered through the 1177 platform operates independently of the EHR. Outcome measures conducted in 1177 must therefore be manually transcribed into the EHR by clinicians. Nationally, patients also have access to their patient records through the 1177 patient portal, which research suggests enhances transparency and engagement (21), although experiences in mental health settings remain mixed, with reported concerns about accuracy and privacy (22). Taken together, these digital systems extend outcome measurement into everyday care, though their impact depends on local workflow integration and data flow between platforms.

Against this background, the present study examined real-world adherence to guideline-concordant outcome measurement in depression treatment across a large regional health system. By comparing traditional treatment modalities with iCBT, we evaluated adherence patterns that highlight systemic challenges in implementing outcome measurement practices that underpin MBC in a context where strong digital health policies coexist with persistent gaps in routine outcome monitoring. Although these digital infrastructures form the broader context for outcome measurement, the present study focused on the documentation of outcome assessments, rather than the scores themselves. Adherence was operationalized as the presence of a recorded PHQ-9 or MADRS entry within defined time windows in the regional EHR.

### 1.1 Study objectives

This study used routinely collected EHR data to evaluate adherence to local clinical guidelines for outcome measurement in depression care in specialized psychiatry in Stockholm. Specifically, we aimed to (1) quantify the proportion of treatment episodes meeting the 60-day and ten-session criteria; (2) compare adherence across treatment modalities, with a particular focus on differences between iCBT, antidepressant pharmacotherapy, and face-to-face psychotherapy; and (3) examine clinician-level factors such as profession and age as predictors of adherence.

The overarching objective was to determine whether long-standing policy expectations have translated into consistent documentation of outcome measures, and to identify structural and organizational factors influencing the real-world implementation of MBC.

## 2 Materials and methods

### 2.1 Study design and setting

This retrospective cohort study (ClinicalTrials.gov identifier: NCT06332261) analyzed routinely collected, de-identified EHR data from five publicly funded specialized adult psychiatric outpatient clinics within Stockholm Health Care Services (SHCS), Region Stockholm, Sweden. The study period covered treatment episodes both initiated and completed between January 1, 2020, and September 30, 2023.

The study protocol, including data extraction and analytic procedures, was reviewed under SHCS’s data-governance framework for research involving patient data. Approval was granted following submission to the registry office and subsequent review by the internal research support unit and the Data Protection Officer.

### 2.2 Study population and cohort selection

The study cohort included adult patients (≥18 years) with a primary diagnosis of depression, defined according to the *International Classification of Diseases, Tenth Revision* (ICD-10; codes F32–F33).

Patients were required to receive treatment from a qualified mental health professional (physician, psychologist, psychotherapist, or social worker) within specialized outpatient psychiatric services.

To ensure a well-defined cohort suitable for analyzing adherence to treatment-specific outcome measurement guidelines, we applied the following exclusion criteria:

1. Combination treatments Combination treatments (e.g., concurrent pharmacotherapy and psychotherapy) were excluded because outcome measurement responsibilities are typically divided between providers, making attribution of adherence ambiguous. Excluding these episodes enabled clearer modality-specific estimates.
2. Non-standard therapeutic modalities (e.g., art therapy, light therapy), for which outcome measurement was not explicitly defined in the clinical process charts.
3. Non-specialist providers (e.g., occupational therapists, physiotherapists) whose primary role was not direct depression treatment.
4. Episodes with incomplete or ambiguous documentation regarding treatment start or end dates, or treatment modality.
5. Low-frequency psychological treatments (e.g., acceptance and commitment therapy, dialectical behavior therapy), which were excluded due to insufficient sample size for meaningful comparison.

These criteria ensured a focus on the most common treatment modalities and enabled robust estimates of adherence to guideline-concordant outcome measurement.

The initial dataset comprised 4105 adult patients. After applying the inclusion and exclusion criteria, the final analytic sample consisted of 2431 distinct treatment episodes involving 2173 unique patients, managed by 506 unique clinicians. Antidepressant (AD) pharmacotherapy was the most common treatment modality (n = 1649; 67.8%), while psychological treatments accounted for 782 episodes (32.2%). Among psychological treatments, iCBT was predominant (n = 648; 82.9% of psychological treatments, 26.6% of all episodes). Face-to-face cognitive behavioral therapy (CBT) accounted for 104 episodes (13.3%), while psychodynamic therapy (PDT; n = 21, 2.7%) and cognitive therapy (CT; n = 9, 1.2%) were less common.

The patient sample was 62.1% female (n = 1349) and 37.9% male (n = 824), with a mean age of 40.4 years (SD = 16.6). The clinician sample was 64.2% female (n = 325) and 35.8% male (n = 181), with a mean age of 43.2 years (SD = 12.3). The clinician cohort included physicians (79.8%, n = 404), psychologists (18.0%, n = 91), psychotherapists (1.2%, n = 6), and social workers (1.0%, n = 5).

### 2.3 Treatment modalities and outcome measurement workflows

The internet-delivered cognitive behavioral therapy (iCBT) platform used within SHCS is implemented through the national 1177 Support and Treatment platform. Within the iCBT platform, patients complete structured self-assessments such as the PHQ-9 at predefined points—typically at screening, pre-, and post-treatment and at follow-up (23). In addition, the self-rated MADRS-S is administered weekly during therapy. The platform provides automated prompts, visual feedback on symptom trajectories, and safety alerts (e.g., flagging high suicide item scores for clinician review). These features are consistent with international best practices for iCBT implementation in routine care (15). However, these data are not automatically transferred to the EHR; instead, clinicians manually document summary information such as PHQ-9 scores in the EHR. This workflow supports measurement-based decision-making but relies on manual documentation, which may vary across clinicians.

In contrast, outcome measurement in traditional face-to-face care is administered through web forms, a digital system integrated with the EHR. Patients complete questionnaires remotely or in clinic, and once the clinician opens the completed form and clicks “Save to journal,” the data are automatically stored as structured text in the EHR. Unanswered or unsaved forms are not recorded. Although some patients receive basic automated confirmation or score summaries, these web forms primarily serve as an administrative tool for structured documentation rather than a real-time feedback system. While this infrastructure enables standardized outcome monitoring, it is not fully automated and lacks the continuous, system-embedded measurement loops characteristic of iCBT workflows.

These two digital workflows—manual documentation from 1177 for iCBT and semi-automated web forms for traditional care—together define the operational context for guideline adherence evaluated in this study.

### 2.4 Definition of guideline-concordant outcome measurement

The local clinical process chart for adult depression treatment (13) specifies that outcome measurement should include at least one PHQ-9 or MADRS assessment within 60 days of treatment initiation (all modalities) and, for psychotherapy, at least one such assessment within the first ten sessions (irrespective of therapy form). The 60-day timeframe reflects the recommendation for follow-up within 1–2 months of treatment initiation. The ten-session criterion corresponds to the clinical guidelines that psychotherapy courses typically involve about 12 sessions.

In accordance with the clinical process chart for adult depression care (12), we applied two adherence criteria:

1. Within 60 days (all modalities): at least one PHQ-9 or MADRS documented in the EHR within 60 calendar days of the recorded treatment start date.
2. Within ten sessions (psychotherapy only): at least one PHQ-9 or MADRS documented within the first ten recorded therapy sessions.

These criteria represent a minimal threshold for guideline adherence. To ensure a fair opportunity for adherence, episodes included in the 60-day analysis were required to have a minimum treatment duration of one month (≥30 days). This requirement reflects updates to the clinical guidelines during the study period, in which the recommended timeframe for follow-up assessment shifted from 1–2 months to a standardized 60-day window. Applying this rule yielded a subsample of 2242 treatment episodes (1586 pharmacotherapy and 656 psychological treatment episodes).

For the ten-session psychotherapy criterion, episodes were required to include at least ten recorded sessions, resulting in a subsample of 482 psychotherapy episodes.

### 2.5 Outcome measures and data extraction

Outcome measurement within SHCS is guided by clinical process charts and quality management protocols. These charts specify the minimum use of validated instruments and require that scores be entered into the EHR as structured text. Units receive monthly feedback reports tracking the proportion of completed web forms documented within 30 days. These indicators are integrated into the local quality management system. However, there are no dedicated audits focusing exclusively on outcome measurement. Instead, adherence is encouraged indirectly through regular feedback reports and quality improvement activities.

The primary assessment instruments specified in the clinical process chart analyzed in this study were the MADRS and the PHQ-9. The MADRS is a 10-item clinician-rated scale with well-established reliability and validity for assessing depressive symptom severity (24, 25). The PHQ-9 is a 9-item patient-reported outcome measure aligned with DSM diagnostic criteria for major depressive disorder and validated for screening, diagnosis, and monitoring (12, 26).

In addition, the self-rated MADRS (MADRS-S) (27) was included in the extraction to capture supplementary information about overall assessment activity, such as the presence of pre- and post-treatment documentation in the EHR. Although MADRS-S is not explicitly required for guideline concordance—since the clinical process charts specify clinician-rated MADRS or PHQ-9—it is frequently used in internet-based treatments and is sometimes manually journaled by clinicians. Accordingly, MADRS-S entries were considered descriptive indicators of real-world measurement practice rather than part of the adherence criteria.

The following data were extracted from the EHR system by authorized SHCS data managers under institutional data governance protocols:

- Patient demographics: age and sex.
- Clinician demographics: age, sex, and professional role.
- Treatment characteristics: treatment modality, start and end dates, and number of sessions.
- Assessment data: dates of administration for all documented MADRS, PHQ-9, and MADRS-S.

Episodes were included only if both a treatment start and termination template were documented in the EHR. When these templates are completed, the system automatically records the corresponding date as the start or end of the episode. To ensure a fair opportunity for guideline adherence, episodes included in the 60-day analysis required a minimum duration of ≥ 30 days, and psychotherapy episodes in the ten-session analysis required at least ten recorded sessions. Adherence was defined as documentation of at least one PHQ-9 or MADRS within the applicable time window (within 60 days for all modalities; within ten sessions for psychotherapy). Missing assessments during the active treatment episode were conservatively coded as non-adherence. Because visits and assessments beyond the recorded end date were not captured, post-termination assessments were not included; this limitation is addressed in the Discussion.

### 2.6 Analysis of pre–post treatment assessments

To provide a broader perspective on real-world outcome measurement beyond strict guideline adherence, we examined the extent to which both pre- and post-treatment assessments were documented. This reflects a core component of MBC, which emphasizes tracking patient-reported and clinician-rated outcomes across the entire treatment episode.

For each episode, we calculated the proportion that included both a pre-treatment and a post-treatment assessment using any of the three instruments—PHQ-9, clinician-rated MADRS, or self-rated MADRS-S. Pre-treatment assessments were defined as those documented within ± 1 week of the recorded treatment start date, and post-treatment assessments as those documented within ± 1 week of the recorded end date. A sensitivity analysis was conducted using an expanded time window of ± 2 weeks to account for minor scheduling variations.

Unlike the guideline-adherence analyses, this analysis included all 2431 treatment episodes, regardless of duration or number of sessions, to provide a comprehensive overview of assessment completeness in routine clinical practice.

### 2.7 Statistical analysis

Descriptive statistics (frequencies, percentages, means, and standard deviations) were used to characterize patients, clinicians, and treatment episodes. Adherence to the outcome-measurement guideline was operationalized as a binary variable. For each treatment episode, adherence was coded as 1 if at least one PHQ-9 or clinician-rated MADRS assessment was documented within the applicable time window (≤ 60 days for all treatment modalities; within 10 sessions for psychotherapy), and 0 otherwise. Episodes that did not meet the minimum treatment length required for meaningful follow-up (≥ 30 days for the 60-day analyses; ≥ 10 sessions for psychotherapy analyses) were excluded. Self-rated MADRS-S assessments were not included, as they are not part of the formal guideline specification and are not automatically transferred to the EHR. Missing values therefore reflect ineligible episodes rather than non-adherence.

Between-group differences in adherence rates across treatment modalities were examined using Pearson’s χ² tests, with Cramer’s *V* reported as the effect-size measure (interpreted using conventional benchmarks for small = 0.10, medium = 0.30, and large = 0.50 associations). Differences in continuous variables (e.g., clinician age) between adherent and non-adherent groups were tested with independent-samples *t*-tests, with Cohen’s *d* as the effect-size estimate. For pre–post assessment completion rates, χ² tests compared completion frequencies across modalities and time windows (± 1 week vs ± 2 weeks). Bonferroni corrections were applied to adjust for multiple pairwise comparisons.

All analyses were conducted using IBM SPSS Statistics 31 (IBM Corp., Armonk, NY). Statistical significance was set at two-sided *p* <.05.

## 3 Results

### 3.1 Treatment episode characteristics

The final study cohort comprised 2431 treatment episodes for depression. Descriptive characteristics—including mean treatment duration, number of visits or sessions, and the number and type of outcome assessments—are summarized in **Table 1**. Antidepressant (AD) treatment accounted for 1649 episodes (67.8%), internet-based CBT (iCBT) for 648 episodes (26.7%), and other traditional psychotherapies (CBT, CT, PDT combined) for 134 episodes (5.5%).

**Table 1:** Treatment characteristics by modality.

Characteristic	AD (n = 1649)	iCBT (n = 648)	CBT (n = 104)	CT (n = 9)	PDT (n = 21)	Total Psychotherapies (n = 782)	Total (All Modalities; n = 2431)
Mean Treatment Length (weeks) (SD) [Min–Max]	47.7 (36.8) [0–191]	9.2 (4.1) [0–39]	13.1 (16.0) [0–84]	28.1 (25.7) [0–77]	17.8 (18.1) [0–70]	10.2 (8.3) [0–84]	35.6 (35.3) [0–191]
Mean Number of Visits/Sessions (SD) [Min–Max]	5.9 (5.9) [1–62]	12.3 (5.8) [1–42]	8.8 (7.6) [1–36]	8.9 (7.2) [1–19]	10.1 (8.6) [1–27]	11.7 (6.3) [1–42]	7.8 (6.6) [1–62]
Mean Number of Assessments During Treatment (SD) [Min–Max]	0.8 (1.5) [0–14]	2.5 (1.9) [0–13]	1.7 (2.7) [0–11]	0.3 (1.0) [0–3]	0.4 (0.9) [0–3]	2.3 (2.1) [0–13]	1.3 (1.8) [0–14]
Mean Number of PHQ-9 Assessments (SD) [Min–Max]	0.2 (0.6) [0–6]	0.8 (0.6) [0–3]	0.5 (1.2) [0–7]	0.1 (0.3) [0–1]	0.1 (0.3) [0–1]	0.8 (0.7) [0–7]	0.4 (0.7) [0–7]
Mean Number of MADRS Assessments (SD) [Min–Max]	0.05 (0.3) [0–4]	0	0	0	0	0	0.03 (0.2) [0–4]
Mean Number of MADRS-S Assessments (SD) [Min–Max]	0.6 (1.2) [0–10]	1.7 (1.7) [0–12]	1.2 (2.6) [0–11]	0.2 (0.7) [0–2]	0.3 (0.8) [0–3]	1.5 (1.8) [0–12]	0.9 (1.5) [0–12]

### 3.2 Guideline-concordant outcome measurement

Among treatment episodes lasting at least one month (n = 2242), 632 episodes (28.2%) included a documented PHQ-9 or clinician-rated MADRS assessment within 60 days of treatment initiation (**Table 2**). Adherence varied sharply by treatment modality. For antidepressant pharmacotherapy, only 10.2% (162 of 1586 episodes) met the 60-day criterion, compared with 71.6% (470 of 656 episodes) in psychological treatments overall. This difference was largely driven by iCBT, where 80.1% (454 of 567 episodes) had a timely assessment, whereas traditional face-to-face psychotherapies such as CBT, CT, and PDT showed substantially lower adherence (18.0%, 16 of 89 episodes), χ²(1, N = 656) = 146.00, *p* <.001, Cramer’s *V* = .47. The association between treatment modality (antidepressant pharmacotherapy vs psychological treatments) and 60-day adherence was large, χ²(1, N = 2242) = 865.14, *p* <.001, Cramer’s *V* = .62. When iCBT episodes were excluded, overall adherence across the remaining modalities declined to 10.6% (178 of 1675 episodes), underscoring the influence of automated digital workflows.

**Table 2:** Guideline-concordant outcome measurement by treatment modality.

Guideline	Treatment Modality	N (Episodes)	Concordant Episodes (n)	Concordance Rate (%)
Within 60 Days	All modalities (≥ 30 days duration)	2242	632	28.2
	Antidepressant pharmacotherapy	1586	162	10.2
	All psychological treatments	656	470	71.6
	- iCBT	567	454	80.1
	- Traditional psychotherapies (CBT, CT, PDT)	89	16	18.0
Within 10 Sessions	All psychotherapies (≥ 10 sessions)	482	354	73.4
	- iCBT	430	342	79.5
	- Traditional psychotherapies (CBT, CT, PDT)	52	12	23.1

PHQ-9 or clinician-rated MADRS within specified time frame.

For the psychotherapy-specific ten-session guideline, 354 of 482 eligible psychotherapy episodes (73.4%) met the criterion (**Table 2**). Adherence was markedly higher in iCBT (79.5%, 342 of 430 episodes) than in traditional psychotherapies (23.1%, 12 of 52 episodes), a highly significant contrast, χ²(1, N = 482) = 75.82, *p* <.001, Cramer’s *V* = .40. Thus, while a majority of psychotherapy episodes achieved at least one outcome assessment within ten sessions, the rate was strongly dependent on modality and the presence of automated assessment routines.

### 3.3 Adherence by clinician profession and age

Adherence varies substantially by clinician profession (**Table 3**). Among physicians—who primarily managed antidepressant treatment—only 10.2% (162 of 1586 episodes) met the 60-day guideline. Psychologists demonstrated much higher adherence, with 470 of 644 episodes (73.0%) concordant with the guideline, whereas adherence among psychotherapists (0 of 5, 0%) and social workers (0 of 7, 0%) was nonexistent. When iCBT episodes were excluded, psychologists’ adherence declined sharply to 20.8% (16 of 77 episodes), underscoring that most adherence occurred within automated iCBT workflows rather than face-to-face practice.

**Table 3:** Guideline-concordant outcome measurement by clinician profession (including and excluding iCBT).

Guideline rule	Clinician profession	Concordant episodes (n/N)	Concordance rate (%)	Excluding iCBT (n/N)	Excl. iCBT rate (%)
Within 60 days (all modalities)	Physician	162/1586	10.2	162/1586	10.2
	Psychologist	470/644	73.0	16/77	20.8
	Psychotherapist	0/5	0	0/5	0
	Social worker	0/7	0	0/7	0
Within 10 sessions (psychotherapy)	Psychologist	354/477	74.2	12/47	25.5
	Psychotherapist	0/3	0	0/3	0
	Social worker	0/2	0	0/2	0

For the psychotherapy-specific ten-session criterion, psychologists again showed the highest adherence, with 354 of 477 episodes (74.2%) meeting the requirement, compared to no documented adherence among psychotherapists (0 of 3) or social workers (0 of 2). However, when iCBT episodes were excluded, psychologists’ adherence fell to 25.5% (12 of 47), and the association between profession and adherence was no longer statistically significant.

Overall, clinician profession was strongly associated with adherence to the 60-day criterion (χ²(3, N = 2242) = 896.14, *p* <.001, Cramer’s V = .63), but this effect diminished markedly once iCBT was excluded (χ²(3, N = 1675) = 10.07, *p* = .018, Cramer’s *V* = .08). For the ten-session rule, the association was small (χ²(2, N = 482) = 13.97, *p* <.001, Cramer’s *V* = .17) and non-significant after excluding iCBT (χ²(2, N = 52) = 1.66, *p* = .44, Cramer’s *V* = .18).

For the 60-day criterion, adherent clinicians were younger than non-adherent clinicians (M = 39.76, SD = 8.82 vs. M = 46.81, SD = 12.92), t(1675.74) = 14.81, *p* <.001, Cohen’s *d* = 0.59. By contrast, for the psychotherapy-specific ten-session criterion there was no significant age difference (M = 37.36, SD = 5.94 vs. M = 37.06, SD = 7.03), t(480) = 0.47, *p* = .64, *d* = 0.05.

### 3.4 Pre-post treatment assessments

Across the full cohort, only 423 of 2431 treatment episodes (17.4%) included both a pre- and post-treatment assessment documented within ± 1 week of the recorded start and end dates. When the observation window was expanded to ± 2 weeks, this proportion increased to 614 episodes (25.3%) (**Table 4A**).

**Table 4a:** Proportion of treatment episodes with documented pre- and post-treatment assessments.

Time window	Treatment modality	N (episodes)	Episodes with pre- & post-assessment (n)	Completion rate (%)
± 1 Week	**All Modalities**	**2431**	**423**	**17.4**
	Antidepressant Treatment	1649	21	1.3
	iCBT	648	377	58.2
	Traditional Psychotherapies	134	25	18.7
± 2 Weeks	**All Modalities**	**2431**	**614**	**25.3**
	Antidepressant Treatment	1649	34	2.1
	iCBT	648	544	84.0
	Traditional Psychotherapies	134	36	26.9

Bold values indicate totals across all treatment modalities.

Completion rates differed sharply by treatment modality. For antidepressant management, only 21 of 1649 episodes (1.3%) met the ± 1-week criterion, rising slightly to 34 episodes (2.1%) under the ± 2-week window. In contrast, adherence was substantially higher in iCBT, with 377 of 648 episodes (58.2%) showing complete pre–post assessments within ± 1 week and 544 episodes (84.0%) within ± 2 weeks. Traditional face-to-face psychotherapies demonstrated intermediate rates, with 25 of 134 episodes (18.7%) meeting the ± 1-week criterion and 36 episodes (26.9%) the ± 2-week criterion.

Completion rates differed significantly across treatment modalities (all *p* <.001), with large effect sizes (Cramer’s *V* = 0.66–0.83). Pairwise comparisons (**Table 4B**) confirmed that iCBT episodes were far more likely to include complete pre–post assessments than either antidepressant treatment or traditional psychotherapy. The magnitude of these differences was large for iCBT vs pharmacotherapy (*V* = 0.68–0.85) and moderate for iCBT vs face-to-face CBT (*V* = 0.25–0.43), underscoring the substantial advantage of digital, workflow-integrated care models. Expanding the observation window modestly improved completion in traditional psychotherapy but had minimal impact in pharmacotherapy, further highlighting structural disparities in measurement integration across care modalities.

**Table 4b:** Pairwise comparisons of pre-post assessment completion by treatment modality.

Comparison	Time window	χ² (df)	P-value	Cramer’s *V*	Completion rates
iCBT vs AD	± 1 week	1051.62 (1)	<.001	.68	58.2% vs 1.3%
iCBT vs AD	± 2 weeks	1656.54 (1)	<.001	.85	84.0% vs 2.1%
iCBT vs CBT	± 1 week	46.81 (1)	<.001	.25	58.2% vs 22.1%
iCBT vs CBT	± 2 weeks	136.86 (1)	<.001	.43	84.0% vs 31.7%
CBT vs AD	± 1 week	173.67 (1)	<.001	.32	22.1% vs 1.3%
CBT vs AD	± 2 weeks	234.27 (1)	<.001	.37	31.7% vs 2.1%

All comparisons were statistically significant after Bonferroni correction (adjusted α = .005). Completion rates refer to the proportion of episodes with both pre- and post-treatment assessments within the specified time window.

## 4 Discussion

This study examined real-world adherence to clinical outcome-measurement guidelines in publicly funded outpatient depression care within Stockholm’s mental health services. Even when defined conservatively—requiring only a single follow-up assessment—fewer than one-third of all treatment episodes met the minimal standard. This shortfall is striking given the extensive evidence base supporting MBC. Landmark trials such as STAR*D (1) and subsequent meta-analyses have shown that systematic symptom monitoring and feedback-informed care improve remission rates, reduce symptom severity, and enhance treatment adherence (2, 5, 6, 8). The persistence of low adherence despite strong policy expectations underscores a central implementation gap: evidence and guidelines alone have not translated into consistent practice.

### 4.1 Treatment modality and structural determinants of adherence

Treatment delivery modality emerged as the strongest determinant of guideline adherence. Adherence was substantially higher in iCBT than in either traditional psychotherapy or pharmacotherapy. Approximately 80% of iCBT episodes fulfilled the 60-day criterion, whereas only about one in five traditional psychotherapy episodes and one in ten pharmacotherapy episodes did so. These differences mirror findings from Steidtmann et al. (9), who showed that embedding automated prompts for patient-reported outcome measures (PROMs) within electronic workflows markedly increased completion rates and clinician engagement. This pattern is consistent with evidence from high-volume digital services where routine measurement is built into the treatment flow and supported by clinical governance and therapist guidance, yielding outcomes at least comparable to face-to-face CBT (16).

The relative advantage of iCBT likely reflects both automation and standardization. Swedish iCBT programs typically follow a twelve-module format delivered over roughly twelve weeks, with built-in symptom assessments and automated reminders that ensure consistent follow-up. The format is time-limited and standardized in both duration and session frequency, providing regular opportunities for outcome measurement. In contrast, face-to-face psychotherapy varies widely in total duration, scheduling regularity, and documentation routines, while pharmacotherapy visits are often spaced weeks apart, reducing opportunities for measurement. Consequently, digital workflows reduce dependence on individual clinician initiative, embedding outcome assessment as a routine component of care rather than an optional administrative task. These structural and organizational features likely account for much of the observed adherence gap.

### 4.2 Distinguishing documentation compliance from true measurement-based care

The outcome-measurement guidelines analyzed here—requiring documentation of a PHQ-9 or clinician-rated MADRS within 60 days or ten sessions—represent a minimal threshold rather than full MBC. Our operationalization therefore captured documentation of at least one follow-up assessment, not continuous feedback-informed monitoring. According to the *APA Professional Practice Guidelines* (28) and de Jong et al. (6), high-quality MBC entails repeated, session-by-session measurement, timely feedback shared with both clinician and patient, and systematic use of these data to guide collaborative treatment decisions. Even perfect adherence to the clinical process chart would thus constitute only a partial form of MBC. The finding that fewer than one-third of episodes met this minimal criterion highlights how far routine practice still is from realizing comprehensive, feedback-informed care.

A critical nuance concerns data capture in digital versus traditional systems. While iCBT formally showed around 80% adherence in EHR-based analyses, this figure likely underestimates actual monitoring. The 1177 platform automatically administers weekly self-rated MADRS-S assessments as part of the treatment flow, but these data are not automatically transferred to the electronic health record (EHR). Analogously, large digital clinics report continuous PROM capture as a core feature of routine care, which supports both quality control and ongoing service improvement (16). This further illustrates how EHR-based audits may underestimate the extent of true monitoring in digitally delivered care when cross-system data integration is limited. As a result, the present dataset captured only manually transcribed assessments. True outcome monitoring within iCBT is therefore higher than indicated by EHR documentation. This distinction illustrates a key point: documentation compliance is not equivalent to measurement activity, and current registry-based methods may systematically underestimate adherence in digitally delivered care. The challenge is thus not only to promote measurement but also to ensure seamless data integration across platforms so that measurement informs both care and quality monitoring.

Beyond guideline-based adherence, the supplementary pre–post analyses (**Tables 4A**, **4B**) provide a broader view of real-world outcome-measurement activity, including self-rated MADRS-S assessments. These analyses revealed the same overall pattern of modality differences, but also allowed for a direct comparison between iCBT and face-to-face CBT—two structurally similar treatment models differing primarily in their digital delivery format. The markedly higher completion rates in iCBT suggest that automation and workflow integration, rather than therapeutic orientation, account for much of the observed advantage. In contrast, other psychotherapy forms showed lower completion rates, reinforcing that systematic measurement is most feasible when embedded in digitally supported, protocol-driven care models.

### 4.3 From digital automation to transferable mechanisms

While iCBT demonstrates how automation and workflow integration can achieve high adherence, these findings should not be interpreted as an argument for replacing traditional care with digital programs. Rather, they reveal transferable mechanisms that can strengthen routine practice: automated scheduling of assessments, seamless EHR integration, and real-time feedback loops. Embedding web-form data capture directly into EHR templates, combined with automated clinician reminders when follow-ups are due, could substantially increase adherence in face-to-face and pharmacotherapy contexts. Similar digital prompts have been shown to increase completion rates and clinician engagement in telehealth environments (9). Translating such principles into hybrid care models could close the implementation gap while maintaining therapeutic flexibility.

### 4.4 Clinician-level correlates and contextual effects

Adherence also varied across clinician characteristics. Psychologists and younger clinicians exhibited higher documentation rates than physicians, psychotherapists, and older clinicians. However, these associations likely reflect contextual rather than individual differences, as the regional iCBT unit—where most clinicians are younger psychologists—operates within a digitally mature environment that automates assessment delivery. This interpretation aligns with previous studies showing that psychologists generally hold more favorable attitudes toward routine outcome monitoring and are more likely to implement feedback systems in practice (5, 29, 30). Effective implementation strategies should therefore integrate both technological and educational approaches—embedding digital prompts and training that address variation in professional background and digital readiness.

Even so, these findings align with broader evidence that psychologists tend to hold more favorable attitudes toward outcome monitoring and are more likely to apply it in practice than other professions (7, 29, 30). Surveys in Norway (30) and the United States (29) have consistently shown that psychologists report higher perceived utility of outcome measures and greater readiness to use them when supported by organizational structures and feedback systems. Such differences likely reflect greater exposure to evidence-based practice, professional norms emphasizing systematic assessment, and higher digital literacy. Effective implementation strategies should therefore address both technological and educational dimensions—ensuring that infrastructure and training work synergistically to promote equitable uptake of measurement-based care across disciplines.

### 4.5 Outcome measurement versus feedback-informed care

While the present study focused on whether outcome measures were documented, the ultimate goal of MBC extends beyond data collection. As emphasized in the APA guidelines (28) and the *Collect–Share–Act* framework (31), MBC becomes clinically meaningful when assessment results are shared and used collaboratively to adjust treatment plans. Previous work by de Jong (6) and others underscores that the clinical benefits of MBC depend on the *use* of feedback to guide decisions, not merely its presence in the record. Future research should therefore examine not only whether assessments occur, but also whether feedback is discussed, documented, and acted upon in practice—particularly in digital and hybrid care pathways.

### 4.6 Limitations

Several limitations warrant consideration. First, reliance on structured EHR data may underestimate actual measurement activity if assessments were conducted but recorded in free text or outside standardized templates. For iCBT, weekly MADRS-S monitoring occurs within 1177 and is not automatically imported into the EHR, further underestimating true monitoring frequency. From an implementation perspective, however, lack of structured documentation still constitutes non-adherence to guideline expectations.

Second, treatment episodes involving both psychotherapy and pharmacotherapy were excluded to ensure that outcome-measurement responsibility could be attributed to a single provider and modality. Although this exclusion enhances interpretability, it limits generalizability to patients receiving integrated or multidisciplinary care, which are common in real-world settings.

Third, missing assessments could reflect either early termination or completed treatments without follow-up documentation. To minimize bias, we required a minimum episode duration of 30 days for the 60-day analyses and at least ten sessions for psychotherapy analyses. Nevertheless, some underestimation of adherence remains possible.

Fourth, our measure of adherence was deliberately conservative, restricted to assessments recorded during active treatment rather than post-termination. This approach aligns with guideline intent, which emphasizes ongoing monitoring to inform active decision-making.

Finally, the findings derive from Stockholm’s publicly funded psychiatric services and may not generalize to regions with different organizational or reimbursement structures. However, international evidence indicates similarly low uptake of MBC across multiple health systems, suggesting that the observed challenges are systemic rather than local (7, 29, 30, 32–35).

### 4.7 Implications for policy and practice

Low uptake of outcome measurement is not unique to Sweden. International studies consistently show that only a minority of clinicians routinely use structured outcome measures in psychiatric care. In the United Kingdom, Gilbody et al. (33) reported that only 11% of psychiatrists regularly used standardized scales, while Macdonald and Fugard (34) noted persistent implementation difficulties despite national initiatives. In the United States, more than 80% of psychiatrists surveyed by Zimmerman and McGlinchey (8) did not employ outcome measures routinely, citing time burden and limited training. Comparable barriers have been documented in Australia (32) and other countries, highlighting the global nature of these challenges.

Recent Australian experience provides a useful international parallel. Large-scale therapist-guided iCBT services such as the MindSpot Clinic have delivered more than a decade of nationwide digital mental health care, integrating continuous outcome monitoring as a core element of routine practice (15, 16). These initiatives demonstrate that high-quality, feedback-informed care can be scaled safely and effectively when supported by structured digital platforms and centralized governance. Their success further underscores that digital infrastructure—not only policy guidelines—is essential to achieving consistent measurement and quality assurance at scale.

Within this landscape, Sweden offers a valuable case study. Despite low adherence in traditional care, the combination of national policy expectations, digital infrastructure, and broad iCBT deployment has enabled substantially higher adherence within digital pathways. These results suggest that sustainable MBC depends less on individual motivation and more on system design—automated, interoperable, and user-friendly digital solutions that make outcome measurement the path of least resistance. At scale, therapist-guided iCBT services have demonstrated how outcome assessment can be automated, feedback-oriented, and clinically governed while using limited therapist time per patient (16). Adapting these mechanisms—automated scheduling, embedded scoring/feedback, and seamless documentation—into traditional care pathways is likely critical for sustainable MBC.

Recent updates to clinical psychiatric guidelines in 2025 now recommend standardized assessments every 2–4 weeks for several diagnostic groups (36). While this development represents a positive step toward more comprehensive MBC, our findings caution that increasing measurement frequency without addressing underlying infrastructure risks widening the gap between policy and practice. Successful implementation will require aligning technological systems, workflow design, and clinician support to ensure that outcome monitoring becomes a natural component of everyday care rather than an added administrative burden.

### 4.8 Future directions

Future implementation research should prioritize digital infrastructures that embed outcome assessment and feedback directly into workflows, such as the Trier Treatment Navigator (TTN) (37) and the Partners for Change Outcome Management System (PCOMS) (38–40). These models demonstrate how continuous feedback can support data-informed decision-making and reduce clinical deterioration. Meta-analyses indicate small-to-moderate effects when feedback is actively used (5). However, therapist engagement, feedback literacy, and organizational support remain critical for sustaining such systems—particularly in complex psychiatric settings requiring integration with EHR-based workflows.

### 4.9 In summary

Minimal outcome measurement remains inconsistently documented in routine depression care, particularly within pharmacotherapy and traditional psychotherapy. Digital platforms such as iCBT demonstrate substantially higher adherence by automating assessment delivery and documentation. These findings highlight that policy expectations alone are insufficient: sustainable implementation of MBC requires integrated digital infrastructure, workflow alignment, and clinician support that together transform outcome measurement from an administrative obligation into an intrinsic component of care.

## Data Availability

The datasets analyzed in this study are not publicly available due to legal and ethical restrictions under Swedish data protection regulations. Requests for access may be directed to the Data Protection Officer at Stockholm Health Care Services (Decision Number: SLSO 2023-3554), and are subject to approval by the relevant data governance authorities. Requests to access the datasets should be directed to gdpr.slso@regionstockholm.se.
